# An automated method to detect and quantify fungiform papillae in the human tongue: Validation and relationship to phenotypical differences in taste perception

**DOI:** 10.1016/j.physbeh.2017.12.003

**Published:** 2018-02-01

**Authors:** Sally Eldeghaidy, Daniel Thomas, Martha Skinner, Rebecca Ford, Timo Giesbrecht, Anna Thomas, Joanne Hort, Susan Francis

**Affiliations:** a Sir Peter Mansfield Imaging Centre, School of Physics and Astronomy, University of Nottingham, UK; b Sensory Science Centre, School of Biosciences, Sutton Bonington Campus, University of Nottingham, Loughborough, UK; c Unilever Research and Development, Port Sunlight, Wirral, Merseyside, CH63 3JW, UK

**Keywords:** Fungiform papillae, PROP, Thermal taster status, Tongue images, Automated counting, Colour segmentation

## Abstract

Determination of the number of fungiform papillae (FP) on the human tongue is an important measure that has frequently been associated with individual differences in oral perception, including taste sensitivity. At present, there is no standardised method consistently used to identify the number of FP, and primarily scientists manually count papillae over a small region(s) of the anterior tip of a stained tongue. In this study, a rapid automated method was developed to quantify the number of FP across the anterior 2 cm of an unstained tongue from high resolution digital images. In 60 participants, the automated method was validated against traditional manual counting, and then used to assess the relationship between the number of FP and taste phenotype (both 6-n-propylthiouracil (PROP) and Thermal Taster Status). FP count on the anterior 2 cm of the tongue was found to correlate significantly with PROP taster status. PROP supertasters (PSTs) had a significantly higher FP count compared with PROP non-tasters (PNTs). Conversely, the common approach used to determine the number of FP in a small 6 mm diameter circle on the anterior tongue tip, did not show a significant correlation irrespective of whether it was determined via automated or manual counting. The regional distribution of FP was assessed across PROP taster status groups. PSTs had a significantly higher FP count within the first centimetre of the anterior tongue compared with the PNT and PROP medium-tasters (PMT), with no significant difference in the second centimetre. No significant relationship was found with Thermal Taster Status and FP count, or interaction with PROP taster status groups, supporting previous evidence suggesting these phenomena are independent. The automated method is a valuable tool, enabling reliable quantification of FP over the anterior 2 cm surface of the tongue, and overcomes subjective discrepancies in manual counting.

## Highlights

• Automatic, rapid quantification of FP across the anterior 2 cm of the tongue
• FP count in small tongue tip areas does not reliably correlate reliably with PROP status.
• Number of FP across the anterior 2 cm of the tongue correlates with PROP status.
• No relationship identified between the number of FP and thermal taster status.
• PROP and thermal taste are independent phenomena.

## 1 Introduction

Human taste papillae on the dorsal surface of the tongue can be classified in three types: fungiform, circumvallate and foliate papillae [19]. Papillae are distributed in a specific pattern. Fungiform papillae (FP) are mushroomed-shaped pink structures located on the anterior two-thirds of the tongue with a higher density being present on the tongue tip compared to other areas of the tongue [26]. Taste buds and mechanoreceptors located in FP are innervated by both gustatory and trigeminal nerve fibres [46]. Therefore, these papillae are thought to be associated with oral sensitivity [24], [33]. The circumvallate papillae are large structures arranged in an arc on the posterior tongue, whilst foliate papillae are clustered at the edges of the tongue. Filiform papillae are numerous threadlike elevations covering most of the tongue surface, however they have no taste function.

The number and shape of FP are highly variable across individuals [23], [25], and in some cases FP number has been associated with individual differences in oral perception. FP density is frequently reported to be higher in individuals who are classified as supertasters of the bitter substance 6-n-propylthiouracil (PROP) compared with PROP medium tasters (PMTs) and PROP non-tasters (PNTs) [1], [2], [9], [10], [28]. However, a number of recent studies have failed to show an association between FP count and PROP rating [11], [14], [21]. Some evidence reports PROP supertasters (PSTs) to be more sensitive to many oral sensations, including prototypical tastants [8], irritants [34] and tactile stimuli [10], than PMTs and/or PNTs. However, others have identified no difference across PTS groups for taste [22], [45] or trigeminal [42], [47] stimuli.

Recently, a taste phenotype termed Thermal Taster Status has been discovered [7], whereby 20 to 50% of individuals (known as thermal tasters (TTs)) perceive a ‘phantom taste’ when the tongue is thermally stimulated (warmed and/or cooled) [1], [15], [48]. Elicited phantom taste sensations include prototypical tastes (sweet, sour, salt, umami, bitter) or other oral sensations (mint, metallic, spicy), with reported sensations varying with the temperature regime of warming or cooling [1], [7], [18], [48]. Previous studies have shown that the anterior tip of the tongue is most sensitive to perceiving temperature and phantom taste [7]. TTs have been reported to have higher sensitivity to pure taste stimuli at supra-threshold levels [7], [15], [16]. The detection threshold of sucrose [31], [48] and difference threshold tartaric acid [31] have been reported to be lower in TTs than thermal non-tasters (TnTs). This heightened sensitivity to pure tastes may be linked to the number of FP located on the tongue tip. Only one previous study has assessed the relationship between FP density and Thermal Taster Status, but reported no significant correlation [1].

The counting of FP in humans is generally achieved using one of the following non-invasive methods; video microscopy [23] which requires high-quality images to be obtained over 30–60 min, and is therefore limited to the research laboratory, or digital photography [40] which provides a rapid method for obtaining high quality images. The current methods for counting FP require staining of the tongue with a dye (blue food colouring), to provide contrast between the FP (which appear pink) and the filiform papillae (coated blue), before a high quality digital image is captured [23]. FP are then manually detected over a small region of the anterior tongue, typically a 5–10 mm circular template placed onto the tongue tip close to midline. Manual counting of FP can be subjective and has been shown to be highly variable across assessors counting the same tongue image [29], however this variability has been shown to significantly decrease with training [29]. To reduce discrepancies between assessors, Nuessle and colleagues [29] proposed the ‘Denver Papillae Protocol’ as a standardised method to characterise FP, this method is based on manual counting papillae in a dyed 10 mm circle of the tongue. Recently, automated methods to detect FP from digital images have been proposed [32], [36], [37], [43], however most of these methods still require staining of the tongue or specialised methods to acquire the image. Overall, the lack of a standardised approach to measuring FP density contributes to discrepancies between studies, especially when exploring variation across taste phenotypes, thus highlighting the need for a robust, accurate method that can be applied outside of the laboratory environment and across large scale studies.

The primary objective of this study was to develop an automated method to detect and quantify distribution and density of FP across the anterior 2 cm of the tongue from a high resolution digital image, and to validate the automated counting against traditional manual counting of FP. Secondary objectives were to use this automated method to locate the area(s) of the tongue with the highest FP count, and to assess the relationship between the number of FP and both PROP taster status and Thermal Taster Status phenotypes.

## 2 Methods

### 2.1 Participants and protocol

The study was approved by the University of Nottingham Medical School Research Ethics Committee. All volunteers gave informed consent before enrolling in the study. Recruitment questionnaires were used to screen out any volunteer who had a known taste dysfunction or were on medication that could affect their taste sensitivity. Sixty healthy volunteers (43 female, age 18–60 years) participated in the study. The 60 participants were chosen from 200 subjects recruited and assessed for taste phenotype, to include equal number across PROP tasters with 20 PNTs, 20 PMTs and 20 PSTs. Across the cohort this resulted in Thermal Taster Status being characterised with 29 TTs, 26 TnTs and 5 uncategorised (Uncat).

Participants attended two separate sessions, each session lasting approximately one hour. During the first session participants were trained to correctly use the general Labelled Magnitude Scale (gLMS), their PROP taster status was determined, and images of the tongue were collected for FP measures. Thermal Taster Status was determined in the second session. A gLMS [17] was used to collect all intensity ratings. The gLMS scale is a category ratio scale used to measure intensity of sensation comprising of categories of ‘no sensation’ (0 mm), ‘barely detectable’ (1.4 mm), ‘weak’ (6 mm), ‘moderate’ (17 mm), ‘strong’ (35 mm), ‘very strong’ (53 mm), and ‘strongest imaginable sensation of any kind’ (100 mm). Participants were familiarised and trained on how to use the gLMS prior to data collection, in order to increase validity [3].

### 2.2 PROP taster classification

PROP taster status was defined based on the intensity ratings of 0.32 mM of PROP (Sigma Aldrich, UK) prepared in deionised water from a reverse osmosis unit, presented and classified according to the method described by Lim et al. [20]. Participants were instructed to apply the PROP solution by rolling a cotton bud (Boots pharmacy, UK) that had been saturated in the solution across the whole tongue for approximately 3 s, and to then rate the intensity of the perceived taste of the PROP solution when it reached its maximum on the gLMS [17]. A 2 min break was given for palate recovery and palate cleansing with deionised water, before a second replicate was conducted. The mean intensity rating from the two replicates were used to classify participants as PNT (< barely detectable), PMT (range between barely detectable and < moderate), or PST (> moderate) [20].

### 2.3 Thermal taster (TT) classification

Thermal Taster Status was assessed using a Medoc Pathway thermode (Medoc, Israel) with intra oral ATS (Advanced Thermal Stimulator), based on the method described by Bajec and Pickering [1]. The thermode (16 × 16 mm square surface) was applied to the anterior tongue tip, the area most responsive to thermal taste [7]. A mouthpiece was engineered to ensure the thermode was in contact with the tongue in a standardised position and with standard pressure across both replicates and assessors. Two warming trials (from 15 °C to 40 °C) and two cooling trials (from 35 °C to 5 °C) were conducted, employing a temperature ramp of 1 °C/s for all trials. Prior to each warming/cooling trial, a baseline temperature of 35 °C was held for 10 s. The warming trial started at 35 °C, cooled to 15 °C and re-warmed to 40 °C at which it was held for 1 s. The cooling trial started at 35 °C, cooled the tongue to 5 °C where it was held at this temperature for 10 s before returning to baseline (35 °C). Warming trials always preceded cooling trials to avoid possible adaptation from the intense, sustained cold stimulation [15]. After the warming trial, participants were told to wait until their tongue temperature and sensation had returned to normal before proceeding onto the next trial, with a minimum of a two-minute break between the trials. If a thermally induced taste was perceived in either the warming or cooling trial, the subjects were asked to indicate the taste quality perceived from a selected list (‘sweet’, ‘salty’, ‘bitter’, ‘sour’, ‘umami’, ‘other please specify’), and rate the perceived intensity on the gLMS. TTs were classified as those individuals who perceived a taste above weak intensity during both replicates of either the warming or cooling trial. TnTs were classified as those individuals who did not perceive a taste on any replicate of any trial. Participants who did not meet the criteria for a clear TT or TnT categorisation were labeled as uncategorised (Uncat).

### 2.4 Acquisition of tongue images

**Fig. 1:**
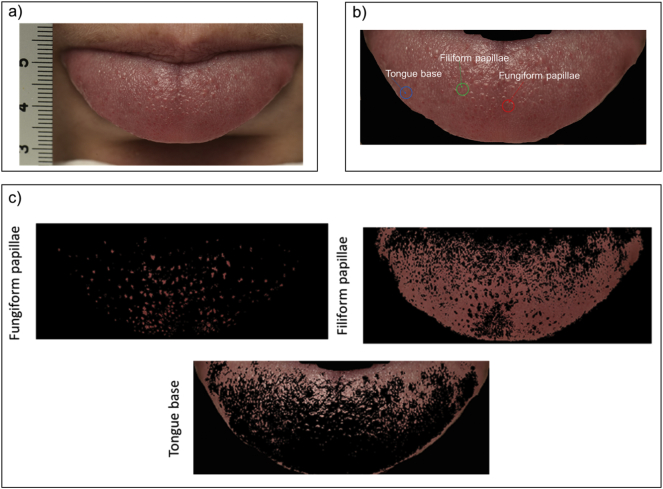
a) Standardised positioning of the tongue for image capturing, with ruler used to scale images, b) An example of manual selection of the three regions of interest (ROIs) - ‘fungiform papillae’, ‘filiform papillae’, and ‘tongue base’, c) Algorithm segmentation of the tongue into ‘fungiform papillae’, ‘filiform papillae’, and ‘tongue base’- based on colour learning.

Digital colour images of the tongue and papillae were collected using a Nikon 4DS camera (16.5 Mpixels, 5000 × 3300 pixels) fitted with 100 mm macro lens. The digital camera was mounted on a column support on a table board equipped with a chinrest to minimise head movement, and to maintain a fixed distance of 35 cm between the participant's tongue and the camera. Studio lighting was used to create a consistent environment to capture the images. Individuals were asked to rest their chin against the chinrest, and adjust their chair height to ensure comfort. In order to generate consistent images, individuals were asked to practice tongue protrusion using a mirror before the image was captured. The tongue needed to be relaxed and held steady by the lips in order to minimise any contrast of light created by folds in the tongue. A ruler was placed next to each participant's tongue to provide a scale of the protruded tongue length, Fig. 1a. The focus was adjusted as necessary to ensure a sharp image with minimal glare. After ensuring sufficient image contrast, three high quality images were collected for each participant for subsequent analysis. All images were resized to only contain the anterior 2 cm of the tongue (Fig. 1b), where FP are most densely located [46].

To allow comparison of the automated counting method with the traditional manual counting procedure within a small ring [23], a 6 mm diameter circle was then placed to the left of the tongue's midline as close to the tongue tip as possible [40]. An image of the tongue was then captured to allow manual counting of FP within this circle using ImageJ software (http://imagej.net/Citing). To assess the effect of using standard dye on the manual count of FP, the tongue tip was then stained with blue food dye using a sterile cotton swab to provide contrast between FP (pink appearance) and the rest of the tongue tissue including filiform papillae (coated blue). An image of the dyed tongue with the 6 mm circle placed on the left of the dyed tongue's midline was then captured to manually count FP in this region.

### 2.5 Fungiform papillae segmentation

An image colour based segmentation algorithm adapted from Rios et al., [36], implemented using the image processing toolbox in Matlab (MATLAB 2016a, The MathWorks, Natick, USA, 2016), was used to detect and quantify FP on the anterior 2 cm of the tongue (scripts provided as Supplementary material).

Here, the algorithm was modified to improve accuracy of automatic detection of FP, and calculate the regional distribution of papillae over the anterior 2 cm of the tongue. The algorithm segments the tongue based on the colour of three manually selected regions of interest (ROIs) (‘fungiform papillae’, ‘filiform papillae’, and ‘tongue base’) to generate three corresponding images based on colour learning, as shown in Fig. 1b and c. FP were first identified as pink, elevated mushroom shape, ‘filiform papillae’ as white threadlike elevations covering the tongue surface, and the ‘tongue base’ as the light pink areas between fungiform and filiform papillae. The three ROIs were then transformed from RGB space to L*a*b space, which is more perceptually uniform than other colour spaces, thus making the colour processing less sensitive to illumination changes. For each ROI, an average colour was determined, and the tongue then segmented according to the nearest neighbour by calculating the Euclidean colour separation between that pixel and the nearest colour. The smallest distance indicates that the pixel most closely matches that colour marker of that ROI. This results in the automatic segmentation of the tongue into three images representing the fungiform papillae, filiform papillae and tongue base, as shown in Fig. 1c. In each image, the area, centroid and boundary were obtained. However, in some cases the colour shade of the FP differs across the tongue (i.e. the tongue tip being more pink than the back of the tongue), or papillae were not individually resolved (i.e. papillae remained interconnected with the ‘tongue base’). To overcome these issues, in our modified analysis method, we allow different areas on the tongue to be analysed independently. The regions to be re-interrogated can be chosen as a rectangular area, and the result of this regional re-analysis is combined with the initial analysis of areas outside this rectangular area.

For each FP detected, its area was automatically measured and the diameter estimated, assuming that FP are circular in shape. The density of FP across the anterior 2 cm of the tongue was computed by dividing the total area of FP by the whole tongue area (sum of ‘fungiform papillae’, ‘filiform papillae’, and ‘tongue base’).

**Fig. 2:**
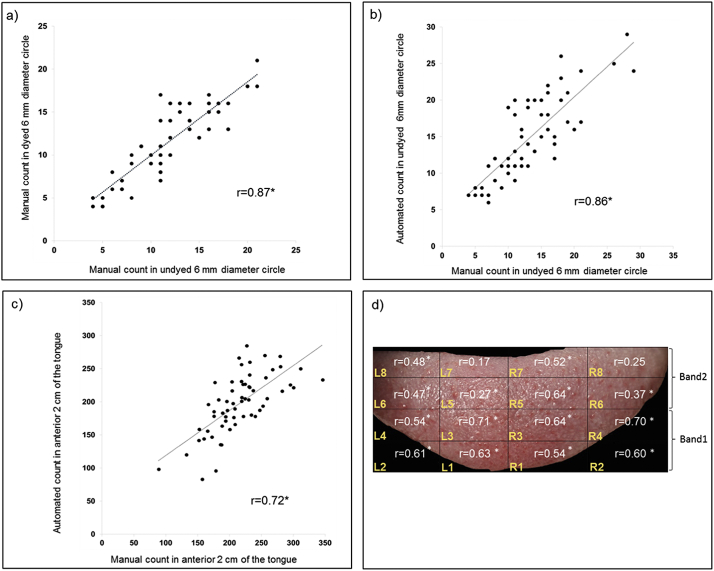
a) A significant correlation between manual fungiform papillae count within a 6 mm circle for the dyed tongue versus undyed tongue, *r* = 0.87, *p* < 0.001. Significant correlations between manual and automated counts for b) the 6 mm circle (*r* = 0.86, p < 0.001) and c) the whole anterior 2 cm region of the tongue (*r* = 0.72, p < 0.001). d) Correlation coefficients (r) between the automated and manual count of papillae in each grid across the anterior 2 cm of the tongue. Grids are numbered from 1 to 4 in Band 1, and from 5 to 8 in Band 2 for the left (L) and right (R) side of the tongue.

Further analysis was conducted using the automated method to determine where within the anterior 2 cm of the tongue FP are most densely distributed. The software divided the digital tongue image through the midline, into left (L) and right (R) sides, enabling assessment of papillae count on each side of the tongue. Second, the software divided the tongue into two 1 cm horizontal bands (Band 1, 1st centimetre, and Band 2, 2nd centimetre). Thirdly, the software divided each side of the tongue into 8 grids, L1-L8 and R1-R8, as illustrated in Fig. 2d.

Note that the automated method is based on a colour based segmentation algorithm and therefore could not be applied to the blue stained tongue images since they do not have sufficient colour contrast to segment the FP from the rest of the tongue image.

### 2.6 Data analysis

#### 2.6.1 Validation of the automated method

First, in Scheme 1, using ImageJ software the number of FP was manually counted in a 6 mm circle of the left side of the tongue on both the dyed and undyed tongue images, following the Denver Papillae Protocol [29]. Second, in Scheme 2, FP were detected using the automated method in the same location as used for the manual count (left side of tongue 6 mm circle) of the undyed images. The diameter of the FP within this 6 mm circle was also determined using both manual and automated methods. A Bland-Altman test was then used to assess the agreement between the manual and automated methods.

A comparison between the manual count (Scheme 3) and automated count (Scheme 4) was also performed on the anterior 2 cm of the tongue using the undyed images. For the manual count (Scheme 3), the number of FP in the anterior 2 cm of the tongue in one of the digital images was assessed by three assessors. An intra-class correlation coefficient (ICC, two-way random, absolute agreement) was computed between the manual count of the FP between the three assessors. For the automated method (Scheme 4), three digital images of the same participant/tongue were processed by one assessor, and the resultant measures of the FP count, density and diameter were averaged across the three images. To assess the reliability of the automatic processing in detecting FP, an ICC was calculated across the three image measures. A Bland-Altman test was then performed between the average manual count (computed across the three assessors) and the average automated count (computed across the three digital images) to assess the agreement between manual and automated FP counts in the anterior 2 cm of the tongue. The automated method was performed prior to the manual count, and the assessor of the manual count was blind to the automated FP count. All assessors were blind to the participant's phenotype for both manual and automated counts. The automated method took approximately 2 min to compute all FP quantitative measures per images, whereas it took approximately 7 min to manually count FP across the anterior 2 cm of the tongue.

#### 2.6.2 Distribution of fungiform papillae across the anterior tongue

The automated method (Scheme 4) was used to determine which band and which grid of the tongue had the highest FP count [40], this was performed independent of taste phenotype. A correlation was also performed between the number of FP within the 6 mm diameter circle, a common size for interrogation in sensory research, in the undyed tongue and the total number of FP on the anterior 2 cm (both side) of the tongue (Scheme 2 and Scheme 4). Further, since the 6 mm circle was placed on the left side of the tongue (mainly covering grids L3 and L5), a correlation was also performed between the number of FP within the 6 mm diameter circle and the total number of FP on the left side of the tongue.

#### 2.6.3 Assessing the relationship between fungiform density and taste phenotype

The relationship between FP count and both PROP and Thermal Taster Status was assessed for the 6 mm circle for both the dyed and undyed tongue, and both manual and automated counts of FP in the anterior 2 cm of the undyed tongue (all Schemes). The regional distribution of FP in each band and grid computed from the automated analysis was also assessed across PROP and Thermal Taster groups. The interaction between PROP and Thermal Taster Status with FP count was also examined, using a 2-way ANOVA (PROP and Thermal Taster Status), and subsequent post-hoc Tukey HSD analysis. In addition, a Spearman correlation was performed between PROP gLMS rating and total FP count across the anterior 2 cm of the tongue as assessed by the automated analysis and manual count.

### 2.7 Statistical analysis

Statistical analyses were performed using SPSS software version 22 (IBM). Correlation between measures was assessed using an intra-class correlation coefficient (ICC, two-way random, absolute agreement) with significance defined as *p* < 0.05. FP count was tested for normality using the Shapiro-Wilk normality test, and if found to be non-normally distributed, a non-parametric correlation analysis was used. A Bland-Altman test and Spearman's correlation coefficient (r) were used to assess the agreement between the manual and automated counts, with significance defined as *p* < 0.01. To assess which bands/grids within the tongue image best correlated with the total number of FP, Spearman's correlation analysis was first performed. As a secondary analysis, a step-wise multiple regression analysis was performed to identify which band/grid within the tongue image best predicted the total number of FP, using the total number of FP on the anterior 2 cm of the tongue as a dependent variable and each band/grid as a predictor. The correlation of PROP intensity rating with FP count was assessed by Spearman's correlation analysis. To assess if the total FP count varies between the left and right sides of the tongue, a paired *t*-test was performed, independent of taste phenotype and across PROP and Thermal Taster Status.

A two-way Analysis of Variance (ANOVA) was used to assess the interaction between PROP and thermal taster on FP count. The interaction between gender and taste phenotypes (PROP and Thermal Taster Status) on the FP count was also assessed using a two-way, repeated measure ANOVA. A Post-hoc Tukey's test was used to assess between subject variables.

## 3 Results

The cohort comprised PROP taster groupings of 20 PNT (with Thermal Taster Status of 8 TTs, 8 TnT, 4 ‘*Uncat*’) 20 PMTs (with Thermal Taster Status of 9 TTs, 11 TnT) and 20 PSTs (with Thermal Taster Status of 12 TTs, 7 TnT, 1 ‘Uncat’). Across the cohort there were 29 TTs and 26 TnTs, with 5 individuals ‘Uncat’.

### 3.1 Validating the automated method

The manual counts of FP within the 6 mm circle on the undyed and dyed images (Scheme 1) were significantly correlated (*r* = 0.87, *p* < 0.001), Fig. 2a, reflecting that dying the human tongue does not have a significant impact on the detection of FP. Significant agreement was found between the number of FP detected from the 6 mm undyed circle using the manual count (Scheme 1) and automated count (Scheme 2), Bland-Altman test *p* < 0.001 and correlation of (*r* = 0.86, *p* < 0.001), Fig. 2b. In addition, a significant correlation was found between the manual count (Scheme 3) and automated count (Scheme 4) on the anterior 2 cm of the tongue (*r* = 0.72, *p* < 0.001), Fig. 2c. A high level of consistency in the number of detected FP was found both between assessors for the manual count (ICC average = 0.92) and between digital images for the automated count (ICC average = 0.89).

The automated FP count in each individual grid significantly correlated with the manual count from the anterior 2 cm of the tongue in all grids except for grids R8 (*r* = 0.25, *p* = 0.056) and L7 (*r* = 0.17, *p* = 0.229), as shown in Fig. 2d.

### 3.2 Distribution of fungiform papillae across the tongue anterior

**Fig. 3:**
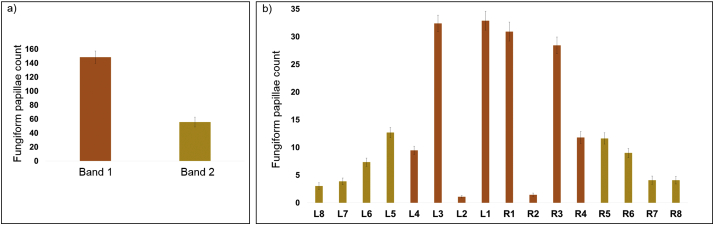
Distribution of fungiform papillae computed using the automated method in a) Band 1 and Band 2, and b) each grid. Error bars indicate standard error (SE).

Across all individuals, independent of taste phenotypes, the mean number of FP detected using the automated method in Band 1, the 1st cm of the anterior 2 cm of the tongue, was 148 (range 53–245) compared to 56 (range 12–151) in Band 2, the 2nd cm of the anterior tongue, Fig. 3a. The distribution of FP in each grid on the left and right side of the tongue are shown in Fig. 3b. Correlation analysis between the total number of FP in the anterior 2 cm of the tongue showed that Band 1 had stronger correlation with the total number of the FP (*r* = 0.76, *p* < 0.001), Table 1, compared with Band 2. A stepwise linear regression analysis showed that Band 1 accounted for 63% of the total number of FP on the tongue, whilst Band 2 accounted for only 37% of the total number of FP.

**Table 1:** Spearman's correlation coefficient (r) of the correlation between total fungiform papillae count and number of papillae in each grid on left and right side of anterior 2 cm of the tongue, with significance *p* < 0.01.

Band number	r	*p*	Grid number	Left		Right	
				r	*p*	r	*p*
Band 1	0.76	< 0.001	1	0.49	< 0.001	0.43	0.001
			2	0.01	0.5	0.29	0.024
			3	0.57	< 0.001	0.65	< 0.001
			4	0.45	< 0.001	0.28	0.03
Band 2	0.44	< 0.001	5	0.36	0.004	0.35	0.006
			6	0.38	0.003	0.194	0.14
			7	0.23	0.075	0.10	0.44
			8	0.35	0.006	0.28	0.032
			6 mm circle	0.39	0.002	–	–

Correlation analysis of the total number of FP in the anterior 2 cm of the tongue detected using the automated method and number of FP in each grid on left and right side of the tongue are shown in Table 1. For the left side, grid L1 and L3 within Band 1 had the highest correlation with the total number of FP in anterior 2 cm of the tongue, with Spearman's correlation coefficient *r* = 0.57 and 0.49, respectively. For the right side, grids R1 and R3 of Band 1 and R5 of Band 2 had the highest correlation with the total number of FP, with Spearman's correlation coefficient *r* = 0.43, 0.65 and 0.35 respectively, independent of taste phenotyping. For the 6 mm diameter circle (placed on the left side of the tongue), only a weak correlation was found with total number of FP in the left anterior 2 cm of the tongue (*r* = 0.32, *p* = 0.013) and strong correlation with the whole anterior 2 cm of the tongue (*r* = 0.39, *p* = 0.002).

Table 2, illustrates the best 5 predictor models for the total number of FP on the anterior 2 cm of the tongue from the stepwise linear regression analysis. The models were calculated based on the linear regression analysis Y = βX_1_ + βX_2_ + βX_3_ + … + constant, where β is the coefficient calculated from the regression analysis and X_n_ the grid number. As shown in Table 2, grid R3 was the best predictor of the total FP count, including grids R6, L1, L3 and L8 into the model increased predictability of the FP count from 0.44 to 0.87.

**Table 2:** Predictor models of total fungiform papillae count on anterior 2 cm of the tongue.

Model		R^2^
1	Y = 2.92R3 + 121.28	0.44
2	Y = 3.11R3 + 3.23R6 + 86.7	0.60
3	Y = 2.31R3 + 3.36R6 + 1.83L3 + 49.2	0.74
4	Y = 2.10R3 + 2.36R6 + 2.02L3 + 3.59L8 + 46.7	0.83
5	Y = 2.08R3 + 2.78R6 + 1.39L3 + 3.72L8 + 1.01L1 + 30.04	0.87

### 3.3 Relationship of taste phenotype with fungiform papillae count

**Fig. 4:**
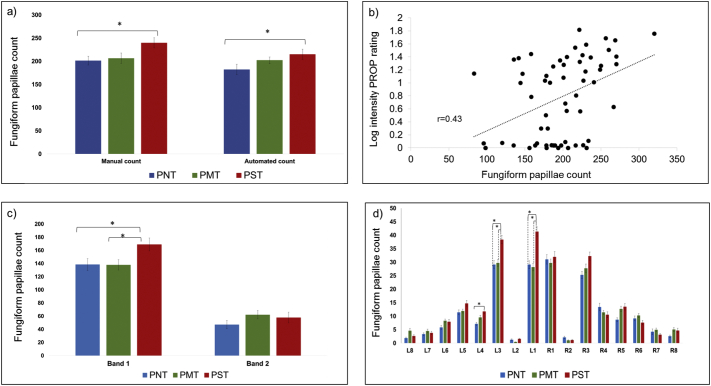
a) Bar chart of the manual and automated fungiform papillae count in anterior 2 cm of the tongue plot for each PROP group. The fungiform papillae count was significantly higher in PSTs compared to PNTs for the manual (*p* = 0.006) and automated (*p* = 0.019) count, as assessed by post-hoc Tukey's analysis. b) The correlation of bitterness rating of PROP with the automated fungiform papillae count on the anterior 2 cm of the tongue. c) Automated fungiform papillae count on the tongue in Band 1 and Band 2 across PROP groups. d) Automated fungiform papillae count on the tongue in each grid across PROP groups. Error bars indicate standard error (SE) and * indicate significance difference.

A two-way AVOVA indicated no significant difference in FP count in the 6 mm circle on the undyed tongue image between PROP or Thermal Taster Status using both manual and automated counts. In contrast, the manual FP count on the anterior 2 cm of the tongue (average across three assessors) showed a significant difference in FP count between PROP groups (ANOVA, *p* = 0.009), Fig. 4a. The post-hoc Tukey's analysis revealed a significant increase in FP count in the PST group (*p* = 0.019) compared with PNT and PMT (*p* = 0.05) groups, whilst no significant difference was found between PMTs and PNTs. The automated FP count on the anterior 2 cm of the tongue (average across three digital images) also showed a significant difference between PROP groups (*p* = 0.005), with a significantly higher count in PSTs compared with PNTs (*p* = 0.006, assessed by post-hoc Tukey's analysis), Fig. 4a. The density of the FP across the anterior of the tongue also showed a significant difference across PROP groups (*p* = 0.011), with a significant increase in PSTs compared with PNTs (*p* = 0.038). No significant difference was found in FP count between Thermal Taster Status groups, nor a significant interaction between PROP and thermal taster groups, for either the manual or automated count. Gender effect on FP count was assessed, but no significant difference was found.

Despite a high level of agreement in the manual count of FP in the anterior 2 cm of the tongue across assessors (ICC = 0.92), results indicate that there were differences between assessors. ANOVA revealed significant differences in FP count between PROP groups was for Assessor 1 (*p* = 0.012) and Assessor 2 (*p* = 0.007), but not Assessor 3 (*p* = 0.056). Tukey post-hoc testing identified a significant increase in FP in PSTs compared to PNT for Assessor 1 (*p* = 0.033) and Assessor 2 (*p* = 0.008), whilst FP count was significantly higher in PSTs than PMTs for Assessor 1 (*p* = 0.027) only. In addition, neither a significant difference between FP count and thermal taster groups, nor a significant interaction between PROP and thermal tasters groups was found for any assessor.

The manual assessment of diameter of FP in the 6 mm circle showed no significant difference in FP size across PROP and thermal taster groups, and no relationship between FP size and count, independent of taste phenotypes was found. The diameter of FP detected with the automated method did not agree/correlate with the manual method and hence was not used for further analysis (see Discussion).

The PROP gLMS intensity rating across all participants (PNT, PMT and PST groups combined *n* = 60) showed a significant correlation (*r* = 0.43, *p* = 0.001) with the automated FP count on the anterior 2 cm of the tongue, Fig. 4b. Table 3 shows the FP count dependence on PROP and Thermal Taster Status. Fig. 4c illustrates the papillae distribution in Bands 1 and 2 of the tongue for PROP groups. ANOVA indicated a significant different in FP count between PROP groups (*p* = 0.004) in Band 1, with PST papillae count being significantly higher than PNTs (*p* = 0.035) or PMTs (*p* = 0.030). No significant difference was found between FP count and thermal taster groups. For Band 2, no significant difference was found between either taste phenotype. Fig. 4d illustrates the papillae distribution in each grids across PROP groups. ANOVA of PROP and thermal taster groups across grids revealed a significant increase in PST FP count in grids L1 and L3 compared with PMT and PNT, whereas in grid L4 a significant difference was found between PSTs and PNTs. Although grid R3 showed a global significant difference in number of FP, no significant difference between PROP and Thermal Taster Status groups was found. A paired-test showed no significant difference between the total number of FP on the right side of the tongue compared with the left side, independent of taste phenotypes. However, PST showed a trend of increasing FP count on the left compared with right side of the tongue, which approached significance (*p* = 0.059). This was driven by differences in grids 1 and 3, in grid 1 there was a greater FP count on the left compared to right side of the tongue in four subjects, leading to a significant difference in the PST group (*p* = 0.02). For grid 3, there were five subjects who had a larger FP count on the left side compared to the right side of the tongue, but this FP count was not significantly different between the sides of the tongue (*p* = 0.06).

**Table 3:** Mean (± SE) and range of fungiform papillae (FP) count in the anterior 2 cm of the tongue (Total fungiform papillae count), Band 1 and Band 2 for PROP (PST, PMT and PNT) and thermal taster (TTs and TnTs) groups.

Group	Total FP count	FP range	Band 1 FP count	Band 2 FP count
PST	231 ± 12	135–396	169 ± 9	62 ± 6
PMT	197 ± 11	96–296	139 ± 8	58 ± 6
PNT	185 ± 10	83–255	138 ± 10	47 ± 8
TTs	206 ± 10	65–233	149 ± 8	57 ± 6
TnTs	203 ± 5	53–207	145 ± 5	58 ± 5

## 4 Discussion

The determination of the number of FP on the human tongue is an important measure in sensory science, food and nutrition research. Most studies count the number of FP on the tongue manually over a small region(s) on the anterior tongue tip. Recently, automated methods to detect FP from digital images have been proposed [32], [36], [37], [43]. In this study, we adapted an algorithm based on colour segmentation previously proposed by Rios and colleagues [36] to detect FP more accurately, and calculate the density and regional distribution of the FP over the tongue. Here, we quantified FP count on the first 2 cm of the anterior part of the tongue, where it has previously been reported that 87% of the FP are located [5], [40].

The automated method demonstrated a fast and accurate detection method to quantify measures of FP. The results showed a strong agreement with laborious manual counting for both a small 6 mm circle (*r* = 0.86) and the anterior 2 cm of the tongue (*r* = 0.72). Manual counting over this size of the tongue can be highly sensitive to subjective discrepancies in FP count, and is time consuming.

The spatial distribution of FP across the anterior 2 cm of the tongue was assessed. The 6 mm diameter circle (covering grids 3 and 5), significantly correlated with the overall FP count on the anterior 2 cm of the tongue. Despite, the results indicating that the 6 mm circle provides a reliable measure of the FP count, we demonstrate that other grids on the tongue show a stronger correlation and provide a better predictor of overall FP number, for example grids L1/R1 and L3/R3 of the tongue. Only two papers have previously assessed the spatial distribution of FP across the tongue and correlated the use of a 6 mm circle with the total number of FP on the tongue [6], [40]. Our results are in agreement with Correa et al., [6] and Shahbake et al., [40] who also demonstrated that locations commonly used to assess papillae count (6 mm circle) are less reliable than other alternative locations. In the present study, Band 1 (first cm) of the anterior tongue was more significantly correlated with the total tongue FP count than Band 2 (second cm). In contrast, Shahbake et al. [40] showed the second cm (the left side of the tongue which was dyed) of the tongue to be more highly correlated to total tongue FP count compared the first cm (although the first cm was still significantly correlated). This difference may arise due to a single side of the tongue being studied in Shahbake et al., together with the greater variability in the shape of the tongue in the first cm across the individuals studied. This could also explain the significantly increased variance explained in the model in Shahbake et al., when using the first cm.

In this current study, the relationship between taste phenotype (PROP and Taster Thermal Status) and FP count was assessed on both a small (6 mm diameter circle) and large (anterior 2 cm) areas of the tongue. In contrast with some previous findings [1], [9], [10], [35], in our study no significant difference was found in FP count in the 6 mm circle across PROP groups, and no significant correlation with PROP intensity ratings. However, the total number of FP on the anterior 2 cm of the tongue significantly correlated with the PROP intensity ratings, and was found to be significantly higher in PSTs compared with PNTs. These findings highlight the importance of performing the FP count on a larger area of the tongue. This study, and other groups, have shown that the number and location of FP on the tongue differ among subjects [5], [24], [25]. A number of recent studies failed to show an association between FP count and PROP rating [11], [14], [21], whereas other studies have identified a significant association [1], [9], [10]. Feeney and Hayes [11] suggested that previously reported associations between FP number and PROP intensity ratings may have been overestimated due to the use of small sample sizes, different areas of the tongue or different methods to detect FP. However, in the largest study to date [14], over 2000 participants (male and female, across all ages) showed no significant association between FP count and PROP rating. In that study, FP were counted within the standard 6 mm diameter circle with the left edge bordering the midline of the tongue. Therefore, we postulate that the lack of association in this large study was due to the use of a small area of the tongue being assessed. This is supported by findings of Delwiche et al., [8], who showed that the correlation of FP number with PROP intensity was dependent on the location on the tongue measured. However, it should be noted that there is likely a complexity of factors involved in PROP and FP count associations. Many other factors (including genetic, demographic, environment) aside from methodological factors can account for the current uncertainty of associations FP count and taste responsiveness. For example, despite Fisher failing to find an association between PROP phenotype and FP count [14], in 2014 the same group [13] did report a significant association between PROP phenotype and genotype. Thus it should be recognised that the data shown here refer to the specific group of subjects studied and larger studies are now required.

Here, we illustrate the regional association of FP count with PROP sensitivity. PSTs show the highest FP count within Band 1 of both the left and right sides of the tongue compared with PNTs and PMTs, Fig. 4. In grids L1 and L3, the papillae count in PSTs was significantly higher than in PMT and PNTs, whilst grid L4 showed a significant increase in PST compared to PNT. Grid R3 showed a global increase in FP but no significant difference between PROP groups. The number and location of FP on the tongue has previously been shown to differ among subjects [5], [24] and show variation in densities [38].

The cohort in the present study was of mixed gender and ethnicity, similar to other populations previously investigated for PROP status [1], [39], [41], with a majority of female participants. Females have been reported in some studies [14], [9], [3], [2] to have a higher number of FP than males and be more likely to be PSTs, whereas other studies have found no difference across gender [4], [44]. Although, the majority of the cohort in this study were female participants, no significant effect of gender on FP count was found when this was included as a factor in the statistical analysis, in line with previous work [4], [44]. No correlation was found between FP diameter and FP count in the 6 mm circle in this study when assessed independent of gender, or in female participants only. The results are in contrast to those of Essick et al. [10], who showed the diameter of FP decreased linearly with increasing FP count in Asian females, with PSTs showing the highest count and smallest diameter compared with PMTs and PNTs. In this current study, the age criteria was 18–60 years, as used in previous similar work [32], [44], however the participants' date of birth was not recorded and so age could not be included as a factor in the statistical analysis. The relationship between FP reduction and ageing has been investigated in a number of studies, while an association has been found in some studies [6], [12], others have failed to show an effect [30]. In addition, the relationship between PROP sensitivity and age has been recently reported to decline in females, whilst no significant difference was observed in males [27]. We acknowledge that the unbalanced age within the PROP groups in this study might have had an effect on the relationship between FP and PROP rating, and a further study balanced for gender and age would be needed to further confirm this observation.

In contrast to PROP groupings, Thermal Taster Status had no association with FP count. Previous work has shown that FP count is associated with increased sensitivity to trigeminal stimulation including fat content [28], oral burn of alcohol [9] and tactical acuity [10]. However, only one study has assessed the relationship between FP density (in a 6 mm diameter circle) and Thermal Taster Status, and also reported no significant correlation [1]. These results add a valuable contribution to the current evidence suggesting that the phenomena of PROP and Thermal Taster Status are likely independent [1], [48].

Most of the recently proposed automated methods [36], [37], [43] to detect FP from digital images include manual selection/detection of FP prior to the automated detection. To our knowledge only one very recent study to date has performed a fully automated method to detect and calculate FP [32]. Although, here we have extended current approaches proposed by Ríos et al. [36] in detecting FP, there are a number of limitations and future directions to consider for this work. The algorithm used to count FP is based on colour segmentation to identify FP on the tongue, thus requires manual selection of a FP, as well as other ROIs for each tongue image. Despite the ability and accuracy of the software to detect FP across the tongue, the FP count at the back of the tongue, in grids L7 and R8 of Band 2 (Fig. 2), did not significantly correlate with the manual count. This could be due to the different contrast of the image at the back of the tongue compared with the anterior part. Another limitation of the software is the measurement of FP diameter, since the automatic measures were not in agreement with the manual diameter measures using ImageJ. Currently, the automatic diameter assessment assumes the FP are circular, and does not take into account the shape nor the roundness of the FP. Recently, two algorithm have been developed to detect FP on the tongue based on papilla shape [43] and roundness [37]. Both methods were developed and coded in Matlab, in future we plan to improve our algorithm by combining colour segmentation method with such methods.

## 5 Conclusion

An algorithm for automatic detection and quantification of FP was extended. The method does not require staining of the tongue with blue dye, and quantifies the FP across a large region of the tongue. A strong correlation was found between the manual and automated count, validating the automated methodology. The number and location of FP on the tongue was found to differ among subjects, highlighting that the use of small region(s) (i.e. a 6 mm diameter circle) near the tip of the tongue, as commonly used in food science research to count FP, may not be a reliable measure, despite the correlation between FP count in the 6 mm diameter circle and the whole anterior 2 cm of the tongue. FP count and density relative to the whole tongue was higher in PROP super-tasters compared with medium and non-tasters. No relationship was found between thermal taster status and FP count, suggesting the phenomena of the PROP and thermal taste are independent. The automated method could provide a valuable tool to food science research, reducing analysis time compared to manual counting, improving detection accuracy, and overcoming subjective discrepancies.

## Funding

This work was supported by the Biotechnology and Biological Sciences Research Council [grant number BB/L000458/1] through an IPA grant with Unilever Research and Development.

## Appendix A Supplementary data

Supplementary figures Image 1
