# Supplementary material for: An automated method to detect and quantify fungiform papillae in the human tongue: Validation and relationship to phenotypical differences in taste perception
Source: Physiol Behav. 2018 Feb 1;184:226–34. doi: 10.1016/j.physbeh.2017.12.003 (PMC5766753; doi:10.1016/j.physbeh.2017.12.003)
Supplement: Supplementary file 1 — Supplementary figures [file mmc1.pdf]

```
clear all
close all
clc

filename = uigetfile({'*.jpg;*.tif;*.png;*.gif','All Image Files';...
    '*.*','All Files' },'Choose Image');

C = imread(filename);

happy=0;

while happy == 0

    close all

    figure(1), imshow(C), title('Tongue')

    prompt={'Enter initial types of tongue to be analysed'};
    name = 'Tongue analysis';
    defaultans = {'3'};
    answer = inputdlg(prompt,name,[1 60],defaultans);
    ncolours = str2double(answer);

    while ncolours <=0 || ncolours > 5

        prompt={'Enter initial types of tongue to be analysed between 1 and
5'};
        name = 'Tongue analysis';
        defaultans = {'3'};
        answer = inputdlg(prompt,name,[1 60],defaultans);
        ncolours = str2double(answer);

    end

    for i = 1:ncolours

        [tongue_pap{i},xi(:,1,i),yi(:,1,i)] = roipoly(C);

    end

    region = [xi,yi];

    [m,no,~] = size(region);

    region_coordinates = region(1:m-1,:,:);

    coord = region_coordinates;

    sample_regions = false([size(C,1) size(C,2) ncolours]);

    for count = 1:ncolours
        sample_regions(:,:,count) = roipoly(C,coord(:,1,count),...
            coord(:,2,count));
```

```
end

cform = makecform('srgb2lab');
lab_tongue = applycform(C,cform);

a = lab_tongue(:,:,2);
b = lab_tongue(:,:,3);
colour_markers = zeros([ncolours, 2]);

for count = 1:ncolours
    colour_markers(count,1) = mean2(a(sample_regions(:,:,count)));
    colour_markers(count,2) = mean2(b(sample_regions(:,:,count)));
end

colour_labels = 0:ncolours-1;
a = double(a);
b = double(b);
distance = zeros([size(a), ncolours]);

for count = 1:ncolours
    distance(:,:,count) = ( (a - colour_markers(count,1)).^2 + ...
        (b - colour_markers(count,2)).^2 ).^0.5;
end

[~, label] = min(distance,[],3);
label = colour_labels(label);
clear distance;

rgb_label = repmat(label,[1 1 3]);
segmented_images = repmat(uint8(0),[size(C), ncolours]);

scrsz = get(groot,'ScreenSize');
figure('OuterPosition',[1 1 scrsz(3) scrsz(4)]);

for count = 1:ncolours
    colour = C;
    colour(rgb_label ~= colour_labels(count)) = 0;
    segmented_images(:,:,:,count) = colour;

    if ncolours <= 4
        subplot(2,2,count)
        imshow(segmented_images(:,:,:,count));
        title(['Type ',num2str(count)])
    elseif ncolours > 4 && ncolours <= 6
        subplot(3,2,count)
        imshow(segmented_images(:,:,:,count));
        title(['Type ',num2str(count)])
    end
end

prompt={'Are you happy with these images? Yes=1 No=0'};
name = 'Repeat?';
defaultans = {'1'};
answer = inputdlg(prompt,name,[1 60],defaultans);
happy = str2double(answer);
```

```
    while happy <0 || happy > 1

        prompt={'Are you happy with these images? Yes=1 No=0'};
        name = 'Repeat?';
        defaultans = {'1'};
        answer = inputdlg(prompt,name,[1 60],defaultans);
        happy = str2double(answer);

    end

end

%% resolve tongue again

% happy=0; this here taken out because sometimes Sally is happy
j = no +1;

while happy == 0

    prompt={'Please enter the number of the image that you wish to resolve
again:  '};
    name = 'Choose image number';
    defaultans = {'1'};
    answer = inputdlg(prompt,name,[1 80],defaultans);
    val1 = str2double(answer);

    while val1 <= 0 || val1 > ncolours

        prompt={'Please enter the number of the image that you wish to
resolve again:  '};
        name = 'Choose image number';
        defaultans = {'1'};
        answer = inputdlg(prompt,name,[1 80],defaultans);
        val1 = str2double(answer);

    end

    image  = segmented_images(:,:,:,val1);

    figure(j); imshow(image)

    prompt={'Enter number of types of tongue to be analysed'};
    name = 'Tongue analysis';
    defaultans = {'2'};
    answer = inputdlg(prompt,name,[1 60],defaultans);
    ncolours1 = str2double(answer);

    while ncolours1 < 2 || ncolours1 >= 3

        prompt={'Enter number of types of tongue to be analysed (2 is
recommended)'};
        name = 'Choose image number';
        answer = inputdlg(prompt,name,[1 80]);
```

```
    ncolours1 = str2double(answer);

end

types = zeros(1,ncolours1);

for i = 1:ncolours1

    [tongue_pap1{i},xi(:,1,i),yi(:,1,i)] = roipoly(image);

    types(i) = i;

end

region = [xi,yi];

[m,~,~] = size(region);

region_coordinates = region(1:m-1,:,:);

coord = region_coordinates;

sample_regions = false([size(image,1) size(image,2) ncolours1]);

for count = 1:ncolours1
    sample_regions(:,:,count) = roipoly(image,coord(:,1,count),...
        coord(:,2,count));
end

cform = makecform('srgb2lab');
lab_tongue = applycform(image,cform);

a = lab_tongue(:,:,2);
b = lab_tongue(:,:,3);
colour_markers = zeros([ncolours1, 2]);

for count = 1:ncolours1
    colour_markers(count,1) = mean2(a(sample_regions(:,:,count)));
    colour_markers(count,2) = mean2(b(sample_regions(:,:,count)));
end

colour_labels = 0:ncolours1-1;
a = double(a);
b = double(b);
distance = zeros([size(a), ncolours1]);

for count = 1:ncolours1
    distance(:,:,count) = ( (a - colour_markers(count,1)).^2 + ...
        (b - colour_markers(count,2)).^2 ).^0.5;
end

[~, label] = min(distance,[],3);
label = colour_labels(label);
```

```
    clear distance;

    rgb_label = repmat(label,[1 1 3]);
    segmented_images1 = repmat(uint8(0),[size(C), ncolours1]);

    figure(j+1)
    scrsz = get(groot,'ScreenSize');
    set(groot,'DefaultFigurePosition',[1 1 scrsz(3) scrsz(4)]);

    for count = 1:ncolours1
        colour = image;
        colour(rgb_label ~= colour_labels(count)) = 0;
        segmented_images1(:,:,:,count) = colour;

        if ncolours1 <= 2
            subplot(2,1,count)
            imshow(segmented_images1(:,:,:,count));
            title(['Type ',num2str(count)])
        elseif ncolours1 > 2 && ncolours1 <= 4
            subplot(2,2,count)
            imshow(segmented_images1(:,:,:,count));
            title(['Type ',num2str(count)])
        end
    end

    prompt={'Are you happy with these images? Yes=1 No=0'};
    name = 'Repeat?';
    defaultans = {'1'};
    answer = inputdlg(prompt,name,[1 60],defaultans);
    happy = str2double(answer);

    while happy <0 || happy > 1

        prompt={'Are you happy with these images? Yes=1 No=0'};
        name = 'Repeat?';
        defaultans = {'1'};
        answer = inputdlg(prompt,name,[1 60],defaultans);
        happy = str2double(answer);

    end

end

%% choose rectangle from original image to replace with rectangle
% from cleaner image

% happy=0;this here taken out because sometimes Sally is happy

while happy == 0

    prompt={'Choose the number of the image that you wish to replace an area
of the original image with'};
    name = 'Choose image';
    defaultans = {'1'};
```

```
    answer = inputdlg(prompt,name,[1 60],defaultans);
    val2 = str2double(answer);

    while val2 <0 || val2 > ncolours1

        prompt={'Choose the number of the image that you wish to replace an
area of the original image with'};
        name = 'Choose image';
        defaultans = {'1'};
        answer = inputdlg(prompt,name,[1 60],defaultans);
        val2 = str2double(answer);

    end

    k = j+2;

    adjusted_image = segmented_images(:,:,:,val1);
    tip = segmented_images1(:,:,:,val2);

    figure(k), imshow(adjusted_image)

    powertoggle = uicontrol('Style','togglebutton','String','Proceed',...
        'Position',[10 10 40 40],'Value',1);

    [m,n,p] = size(adjusted_image);

    rect = imrect(gca);

    while get(powertoggle,'Value')==get(powertoggle,'Max')

        pause(0.01)

    end

    pos = getPosition(rect);
    %  [xmin ymin width height]

    xmin = round(pos(1));
    ymin = round(pos(2));

    width = round(pos(3));
    height = round(pos(4));

    xmax = xmin + width;
    ymax = ymin + height;

    if xmin<0 || xmax>n || ymin<0 || ymax>m
        disp('not okay')
    else
        disp('okay')
    end

    while xmin<0 || xmax>n || ymin<0 || ymax>m
```

```
    set(powertoggle,'Value',1);

    while get(powertoggle,'Value')==get(powertoggle,'Max')

        pause(0.01)

    end

    pos = getPosition(rect);

    xmin = round(pos(1));
    ymin = round(pos(2));

    width = round(pos(3));
    height = round(pos(4));

    xmax = xmin + width;
    ymax = ymin + height;

    pause(0.01)

end



%% splice image together for papillae

for i = ymin:ymax
    for ij = xmin:xmax

        adjusted_image(i,ij,1) = tip(i,ij,1);
        adjusted_image(i,ij,2) = tip(i,ij,2);
        adjusted_image(i,ij,3) = tip(i,ij,3);

    end
end

scrsz = get(groot,'ScreenSize');
figure(k+1)
set(groot,'DefaultFigurePosition',[1 1 scrsz(3) scrsz(4)]);

subplot(2,1,1), imshow(adjusted_image)
subplot(2,1,2), imshow(C);

prompt={'Are you happy with this image? Yes=1 No=0'};
name = 'Repeat?';
defaultans = {'1'};
answer = inputdlg(prompt,name,[1 60],defaultans);
happy = str2double(answer);

while happy <0 || happy > 1
```

```
        prompt={'Are you happy with this image? Yes=1 No=0'};
        name = 'Repeat?';
        defaultans = {'1'};
        answer = inputdlg(prompt,name,[1 60],defaultans);
        happy = str2double(answer);

    end

end


%% rejected papillae leftover from the rectangle, treated as base tongue

if(~happy)
    for i = 1:ncolours1

        otherchoices(i) = i;

    end

    otherchoices(val2) = 0;

    [row,col,v] = find(otherchoices);

    tipextra = segmented_images1(:,:,:,v);

    matrix = zeros(m,n,p,'uint8');

    j = k+2;

    for i = ymin:ymax
        for ij = xmin:xmax

            matrix(i,ij,1) = tipextra(i,ij,1);
            matrix(i,ij,2) = tipextra(i,ij,2);
            matrix(i,ij,3) = tipextra(i,ij,3);

        end
    end

end
%% thresholding

if(~happy)
    segmented_images(:,:,:,val1) = adjusted_image;
else
    [m,n,p,n4] = size(segmented_images);
    matrix = zeros(m,n,p,'uint8');
    val1=1; % Default value if it all works!
end

happy = 0;
```

```
% segmented_images(:,:,:,val1) = adjusted_image;
% keyboard
scrsz = get(groot,'ScreenSize');
figure('OuterPosition',[1 1 scrsz(3) scrsz(4)]);

all_images = zeros(m,n,p,(ncolours+1),'uint8');

for i = 1:ncolours

    all_images(:,:,:,i) = segmented_images(:,:,:,i);

end

all_images(:,:,:,i+1) = matrix;


if ncolours <=3 && ncolours > 0

    for i = 1:ncolours+1
        subplot(2,2,i), imshow(all_images(:,:,:,i))
        title(['Type ',num2str(i)]);
    end

elseif ncolours > 3 && ncolours <= 5

    for i = 1:ncolours+1
        subplot(3,2,i), imshow(all_images(:,:,:,i))
        title(['Type ',num2str(i)]);

    end

end


while happy==0

    k = j+1;

    num = 1;

    prompt={'Please enter a threshold value for the minimum size of papillae
in pixels'};
    name = 'Threshold?';
    defaultans = {'40'};
    answer = inputdlg(prompt,name,[1 80],defaultans);
    threshold = str2double(answer);

    for i = 1:num

        prompt={'Please enter the number of the image that you wish to
threshold from most recent subplot  '};
        name = 'Choose image number';
        defaultans = {'1'};
```

```
        answer = inputdlg(prompt,name,[1 80],defaultans);
        val = str2double(answer);

        while val > ncolours+1 || val <= 0

            prompt={'Please enter the number of the image that you wish to
threshold  '};
            name = 'Choose image number';
            defaultans = {'1'};
            answer = inputdlg(prompt,name,[1 80],defaultans);
            val = str2double(answer);

        end

        choices(i) = val;

        figure(k)

        rgb_papillae = all_images(:,:,:,val);
        p_gray = rgb2gray(rgb_papillae);
        p_thes =bwareaopen(p_gray,threshold);
        subplot(num,1,i), imshow(p_thes)

    end

    prompt={'Are you happy with this? Yes=1 No=0'};
    name = 'Repeat?';
    defaultans = {'1'};
    answer = inputdlg(prompt,name,[1 60],defaultans);
    happy = str2double(answer);

end

%%
j = k;

for i = 1 : ncolours+1
    rgb_papillae = all_images(:,:,:,i);
    p_gray= rgb2gray(rgb_papillae);
    p_thes=bwareaopen(p_gray,threshold);

    [B,L] = bwboundaries(p_thes,'noholes');

    hold on
    for k = 1:length(B)
        boundary = B{k};

        stats = regionprops(L,'Area');
        statsp_c = struct2cell(stats);

        values{i} = statsp_c;

    end
```

```
end

%% calculating areas and densities

species_number = zeros(ncolours+1,1);

% for i = 1:ncolours+1 -- check in correction mode!
for i = 1:size(values,2)

    z = values{1,i};
    zdouble = cell2mat(z);
    sumz(i) = sum(zdouble);
    [~,species_number(i)] = size(zdouble);

end
for i = 1:num

    disp(['Total number of papillae is
',num2str(species_number(choices(i)))]);

end

totalpixels = sum(sumz);
[m1,n1] = size(choices);

for i = 1:n1

    density(i) = sumz(choices(i))/totalpixels;

end

pap_density = sum(density);

disp(['The papillae density is ',num2str(pap_density)]);


% up to 26/07/16 fixed little bugs and adjusted imrect code

%% Areas of the papillae are given in the values{1,i} cell

% assume circular, therefore A = pi * r^2

pap_areas = cell2mat(values{1,val1});

[~,n2] = size(pap_areas);

pap_radii = sqrt(pap_areas/pi);

pap_diameters = pap_radii*2;

figure(j);
```

```
mvp = ceil(max(pap_diameters));

x = linspace(0, mvp, 81);

subplot(2,1,1)
hist = histogram(pap_diameters,x);
xlabel('diameters');
ylabel('frequency');
title('Diameters - whole tongue')

mvp1 = ceil(max(pap_areas));
x1 = linspace(0,mvp1,81);

subplot(2,1,2)
hist1 = histogram(pap_areas,x1);
title('Areas - whole tongue')

counts = hist.Values;

happy = 0;

while happy ==0

    prompt={'Are you happy with this image? Yes=1 No=0'};
    name = 'Repeat?';
    defaultans = {'1'};
    answer = inputdlg(prompt,name,[1 60],defaultans);
    happy = str2double(answer);

    while happy <0 || happy > 1

        prompt={'Are you happy with this image? Yes=1 No=0'};
        name = 'Repeat?';
        defaultans = {'1'};
        answer = inputdlg(prompt,name,[1 60],defaultans);
        happy = str2double(answer);

    end

end

%% GriddingCode2.m

papillae_image = all_images(:,:,:,1);

num1 = 2;

image = zeros(m,n,p,num1,'uint8');
imageC = zeros(m,n,p,num1,'uint8');


value1 = round(linspace(1,n,num1+1));
```

```
k = j;
k = k+1;
figure(k);

for i = 1:num1

    for j = value1(i):value1(i+1)

        image(:,j,:,i) = papillae_image(:,j,:);
        imageC(:,j,:,i) = C(:,j,:);

    end

    subplot(num1,1,i), imshow(image(:,:,:,i));
    title(['Side ',num2str(i)]);

end

%%
%
prompt={'Choose number of vertical sections'};
name = 'Number of sections';
defaultans = {'4'};
answer = inputdlg(prompt,name,[1 60],defaultans);
num2 = str2double(answer);

while num2 <1 || num2 > 8

    prompt={'Choose number of vertical sections'};
    name = 'Number of sections';
    defaultans = {'4'};
    answer = inputdlg(prompt,name,[1 60],defaultans);
    num2 = str2double(answer);

end

prompt={'Choose number of horizontal subsections'};
name = 'Number of subsections';
defaultans = {'2'};
answer = inputdlg(prompt,name,[1 60],defaultans);
input1 = str2double(answer);

while input1 <1 || input1 > 5

    prompt={'Choose number of horizontal subsections'};
    name = 'Number of subsections';
    defaultans = {'2'};
    answer = inputdlg(prompt,name,[1 60],defaultans);
    input1 = str2double(answer);

end


%%
```

```
sides = 2;

image2 = zeros(m,n,p,num2,sides,'uint8');
image2C = zeros(m,n,p,num2,sides,'uint8');

value2 = round(linspace(1,m,num2+1));

for input = 1:sides

    for i = 1:num2

        for j = value2(i):value2(i+1)

            image2(j,:,:,i,input) = image(j,:,:,input);
            image2C(j,:,:,i,input) = imageC(j,:,:,input);

        end

    end

    imageR21 = zeros(m,n,p,input1,sides,'uint8');
    imageR21C = zeros(m,n,p,input1,sides,'uint8');

    for i = 1:num2

        [~,col,~] = find(image2C(:,:,1,i,input));


        left(input) = min(col);
        right(input) = max(col);

        xpap(i,:,input) = round(linspace(left(input),right(input),input1+1));

    end

end
    imagesgrid = zeros(m,n,p,input1*num2,sides,'uint8');
    imagesgridC = zeros(m,n,p,input1*num2,sides,'uint8');


for input = 1:sides
    counter = 0;
    k = k+1;
    figure(k);

    for i = 1:num2

        y = xpap(i,:,input);

        imageR21 = zeros(m,n,p,input1,sides,'uint8');
        imageR21C = zeros(m,n,p,input1,sides,'uint8');
```

```
        for l = 1:input1

            for j = y(l):y(l+1)

                imageR21(:,j,:,l,input) = image2(:,j,:,i,input);
                imageR21C(:,j,:,l,input) = image2C(:,j,:,i,input);
            end

            counter = counter+1;

            imagesgrid(:,:,:,counter,input) = imageR21(:,:,:,l,input);
            subplot(num2,input1,counter),
imshow(imagesgrid(:,:,:,counter,input))
            title(['Side ',num2str(input),' Grid ',num2str(counter)])

            imagesgridC(:,:,:,counter,input) = imageR21C(:,:,:,l,input);
        end

    end

    [m,n,p,number,si] = size(imagesgrid);

    for i = 1 : number
        rgb_papillae = imagesgrid(:,:,:,i,input);
        p_gray= rgb2gray(rgb_papillae);
        p_thes=bwareaopen(p_gray,threshold);

        [B,L] = bwboundaries(p_thes,'noholes');

        for ki = 1:length(B)
            boundary = B{ki};

            stats = regionprops(L,'Area');
            statsp_c = struct2cell(stats);

            papvalues{input,i} = statsp_c;
        end

    end

end

%%

sumzgrid = zeros(sides,number);
species_number_grid = zeros(sides,number);
mean_radii = zeros(sides,number);

for input = 1:sides

    for i = 1:number

        zgrid = papvalues{input,i};
```

```
        zdoublegrid = cell2mat(zgrid);
        sumzgrid(input,i) = sum(zdoublegrid);
        [~,species_number_grid(input,i)] = size(zdoublegrid);

        disp(['Number of papillae in area ',num2str(i),' on side
',num2str(input),' is ',num2str(species_number_grid(input,i))]);

        pap_radii_grid = sqrt(zdoublegrid/pi);
        pap_diameters_grid = pap_radii_grid*2;

        diameters{input,i} = pap_diameters_grid;

        mean_radii(input,i) = mean(pap_radii_grid);

        disp(['Mean radius in area ',num2str(i),' on side ',num2str(input),'
is ',num2str(mean_radii(input,i))]);

    end

        [row2,col2] = find(species_number_grid(input,:));
        infocol{input} = col2;

        [~,max1(input)] = size(infocol{1,input});

end

gridsizes = zeros(2,counter);
densitygrid = zeros(2,counter);

for input = 1:sides

for i=1:counter

    kC{input,i} = find(imagesgridC(:,:,1,i,input));

[gridsizes(input,i),~] = size(kC{input,i});

densitygrid(input,i) = sumzgrid(input,i)/gridsizes(input,i);

end

end

%%

% want to save densitygrid variable

for input = 1:sides


         sizeplot = max1(input);
%      xcol = infocol{1,input};
```

```
        inf = infocol{1,input};


    for i  = 1:sizeplot

        histgrid = ceil(max(diameters{input,inf(i)}));
        x = linspace(0, histgrid, 15);

%         histarea = ceil(max(cell2mat(papvalues{input,i})));
%         x1 = linspace(0, histarea, 15);

%         inf = infocol{1,input};
%         info = inf(i);

        figure;
        hist = histogram(diameters{input,i},x);
        xlabel('diameters');
        ylabel('frequency')
        title(['Side ',num2str(input),' Grid ',num2str(inf(i))])



%         figure;
%         hist1 = histogram(papvalues{input,i},x1);
%         xlabel('Areas');
%         ylabel('frequency')
%         title(['Side ',num2str(input),' Grid ',num2str(info)])

    end
end

%%

save('pqfile.mat','papvalues','diameters','densitygrid','sumzgrid','gridsizes
')
```
